# Improved virologic outcomes over time for HIV-infected patients on antiretroviral therapy in a cohort from Rio de Janeiro, 1997–2011

**DOI:** 10.1186/1471-2334-14-322

**Published:** 2014-06-11

**Authors:** David A Martin, Paula M Luz, Jordan E Lake, Jesse L Clark, Valdilea G Veloso, Ronaldo I Moreira, Sandra W Cardoso, Jeffrey D Klausner, Beatriz Grinsztejn

**Affiliations:** 1 University of California, Los Angeles, Los Angeles, USA; 2 Fundação Oswaldo Cruz, Instituto de Pesquisa Clínica Evandro Chagas, HIV/AIDS Clinical Research Center, Rio de Janeiro, Brazil

**Keywords:** Antiretroviral therapy, Cohort studies, Brazil, Viral load, Adherence

## Abstract

**Background:**

Previous cohort studies have demonstrated the beneficial effects of antiretroviral therapy (ART) on viral load suppression. We aimed to examine the factors associated with virologic suppression for HIV-infected patients on ART receiving care at the Evandro Chagas Clinical Research Institute, Oswaldo Cruz Foundation in Rio de Janeiro, Brazil.

**Methods:**

HIV-1 RNA levels and CD4+ T-cell counts at the date closest to midyear (1 July) were evaluated for 1,678 ART-naïve patients ≥18 years of age initiating ART between 1997 and 2010. The odds ratios (OR) and 95% confidence intervals (CI) for having an undetectable viral load (≤400 copies/mL) were estimated using generalized estimating equations regression models adjusted for clinical and demographic factors. Time-updated covariates included age, years since HIV diagnosis, hepatitis C diagnosis and ART interruptions.

**Results:**

Between 1997 and 2011, the proportion of patients with an undetectable viral load increased from 6% to 78% and the median [interquartile range] CD4+ T-cell count increased from 207 [162, 343] to 554 [382, 743] cells/μL. Pre-treatment median CD4+ T-cell count significantly increased over the observation period from 114 [37, 161] to 237 [76, 333] cells/μL (p < .001). The per-year adjusted OR (aOR) for having undetectable viral load was 1.18 (95% CI = 1.16-1.21). ART interruptions >1 month per calendar significantly decreased the odds [aOR = 0.32 (95% CI = 0.27-0.38)] of having an undetectable viral load. Patients initiating on a protease inhibitor (PI)-based first-line regimen were less likely to have undetectable viral load [aOR = 0.72 (95% CI = 0.63-0.83)] compared to those initiating on a non-nucleoside reverse transcriptase inhibitor (NNRTI)-based regimen.

**Conclusions:**

Our results demonstrate significant improvements in virologic outcomes from 1997 to 2011, which persisted after adjusting for other factors. This may in part be due to improvements in care and new treatment options. NNRTI- versus PI-based first-line regimens were found to be associated with increased odds of having an undetectable viral load, consistent with previous studies. Treatment interruptions were found to be the most important determinant of not having an undetectable viral load. Studies are needed to characterize the reasons for treatment interruptions and to develop subsequent strategies for improving adherence to ART.

## Background

Universal access to antiretroviral therapy (ART) for human immunodeficiency virus (HIV) infection was first established in Brazil by a federal decree issued in 1996. Over time, the Ministry of Health has continued efforts to expand HIV testing and treatment programs. CD4+ T-cell count thresholds for treatment initiation have increased over time and newer first-, second- and salvage-line antiretroviral drugs with greater efficacy and lower toxicity profiles have been introduced within the National AIDS Program. The early introduction of viral load monitoring within the National ART guidelines in Brazil has also allowed for earlier detection of virologic failure and subsequent treatment modifications. Data from previous studies conducted in both high- and low-resource settings have validated the impact of ART on decreasing morbidity and mortality, and achieving virologic suppression and immune system reconstitution for HIV-infected persons [1-10].

Given the importance of maintaining virologic suppression for reducing the risk of clinical progression of HIV and ultimately death, as well as decreasing HIV transmissibility [11], studies have analyzed the association between clinical and demographic factors and HIV-1 RNA levels to determine factors associated with virologic suppression [5,9]. Data from the Swiss HIV Cohort found treatment interruptions and poor adherence to be the most predictive factors of not maintaining virologic suppression [5]. In Brazil, there are published data regarding predictors of virologic response after ART initiation; however, there are limited data regarding population-level long-term response to ART [6].

This analysis aimed to examine the virologic and immunologic response to HIV treatment, including factors associated with virologic suppression for HIV-infected patients on ART receiving care at the Evandro Chagas Clinical Research Institute (IPEC) of the Oswaldo Cruz Foundation (Fiocruz) in Rio de Janeiro, Brazil from 1997 to 2011. Our analysis, though not representative of the overall Brazilian HIV-infected population, reflects the group-level effects of provision of universal access to ART and continued efforts to improve care and treatment programs both at the national and local level.

## Methods

### Study population

Data were obtained from the longitudinal, clinical database of HIV-infected patients receiving care at IPEC in Rio de Janeiro, Brazil. IPEC has been providing care to HIV-infected patients since 1986. The clinical database was established in 1998 and has since been updated regularly using outpatient and inpatient medical records, and laboratory results. ART regimen data, including drug type, dose and prescription dates, is documented by the medical provider in the medical record. Professional abstractors review all patient records on a continuous basis and record the information onto standardized forms, which are subsequently scanned for inclusion in the clinical database.

We analyzed data for HIV-infected patients greater than or equal to 18 years of age who initiated highly-active ART for the first time during the period from January 1, 1997 through December 31, 2010. Patients initiating ART prior to enrollment in the IPEC clinical cohort were excluded from the analysis. Only patients initiating ART on a non-nucleoside reverse transcriptase inhibitor- (NNRTI) or protease inhibitor- (PI) based regimen were included. NNRTI-based regimens were defined as a NNRTI or NNRTI and PI, plus nucleoside reverse transcriptase inhibitor (NRTI) backbone. PI-based regimens were defined as a PI plus NRTI backbone only. Patients initiating on neither NNRTI- or PI-based first-line regimens were too few (n = 28) to include in our statistical analysis and were therefore excluded. End of follow-up was determined by either year of death for patients with a documented date of death or until the last year of the period of analysis, December 31, 2011.

### Variables of interest

HIV-1 RNA levels and CD4+ T-cell counts at the date closest to midyear (1 July) were evaluated for all patients initiating ART between 1997 and 2011. Only HIV-1 RNA levels and CD4+ T-cell counts occurring greater than or equal to 90 days after the initiation of ART were included in the analysis to allow time for virologic response to treatment. The outcome of interest was undetectable viral load, defined as ≤400 copies/mL to account for less sensitive virus detection thresholds of older testing platforms included in the analysis.

Antiretroviral regimens were classified by drug mechanism of action. No distinction was made for drug generations or agents within the same drug class. Individual ART regimens per calendar year were determined by choosing the regimen with the greatest number of documented months per calendar year. Treatment interruptions per calendar year were defined as the sum of all the periods of time with no documented ART regimen in the patient’s clinical chart, rounded to the nearest month, and not necessarily occurring consecutively.

Demographic characteristics were also assessed, which included: age, gender, race, years of formal schooling, self-reported risk group and date of HIV diagnosis. Race was categorized as White and Non-White. HIV risk group was hierarchically categorized as Injection Drug Users (IDU), Men who Have Sex with Men (MSM), Heterosexual Men, Heterosexual Women, and Other. The “Other” risk group category includes hemophiliacs, occupational exposures, and transfusions. Covariates of interest included pre-treatment CD4+ T-cell count, defined as CD4+ T-cell count occurring within 180 days prior to ART initiation, and lifetime occurrence of hepatitis C diagnosis, defined as the presence of hepatitis C antibodies. Pre-treatment CD4+ T-cell counts were categorized as follows: ≤200, 201–350, 351–500, >500 and missing. Pre-treatment viral load data were not universally available.

### Statistical methods

HIV-1 RNA levels were stratified as ≤400, 401–1,000, 1,001-100,000 and ≥100,001 copies/mL. Median and interquartile ranges (IQR) of CD4+ T-cell counts were computed for each calendar year. Time trends for categorical and continuous variables were tested using the Chi-square and Kruskal-Wallis test respectively, assuming a significance level of p < 0.05.

Unadjusted and adjusted logistic regression models were used to describe the association of clinical and demographic factors with the odds of having an undetectable viral load. Generalized estimating equations (GEE) were used to account for repeated measures per patient. Time trends were analyzed by using calendar year as a continuous variable. The following fixed covariates were included in the model: sex, race, education level, risk group, pre-treatment CD4+ T-cell count and initial ART regimen. Missing values for race and education categories for 5 and 8 patients, respectively, were imputed based on sample distribution. Time-updated covariates included age in years (strata: <30, 30–39, 40–49 and ≥50), years since HIV diagnosis, ART interruptions (categorized as ≤1 month versus >1 month), and hepatitis C diagnosis (history of positive anti-HCV antibody test). ART interruptions were categorized because the variable was not linearly associated with the outcome when treated as a continuous variable (data not shown). Given that the duration of ART interruption did not necessarily represent a continuous interval of time, the variable was categorized as interruptions ≤1 month versus >1 month per calendar year. ART regimen was not included as a time-updated covariate due to limitations in the formatting of the longitudinal dataset. Patients with missing mid-year viral load outcomes were excluded from the logistic regression analysis for the years where they had no viral load outcomes. A sensitivity analysis assuming that all missing virologic outcomes were detectable was performed. Analyses were done using R-software (version 2.15.1).

### Ethical approval

This study was approved by the institutional review board of IPEC. Written informed consent for enrollment in the cohort was obtained from all patients. Retrospective analysis of previously collected data delinked from personal identifying information was considered exempt by the UCLA IRB.

## Results

### Demographic and clinical time trends

A total of 1,678 patients initiated ART from 1997 to 2010 and contributed at least one viral load outcome to the descriptive and logistic regression analysis. There were a total of 8,870 years of follow-up included in the observation period. The median [IQR] number of months of follow-up per patient was 47 [29, 79] months.

**Figure 1:**
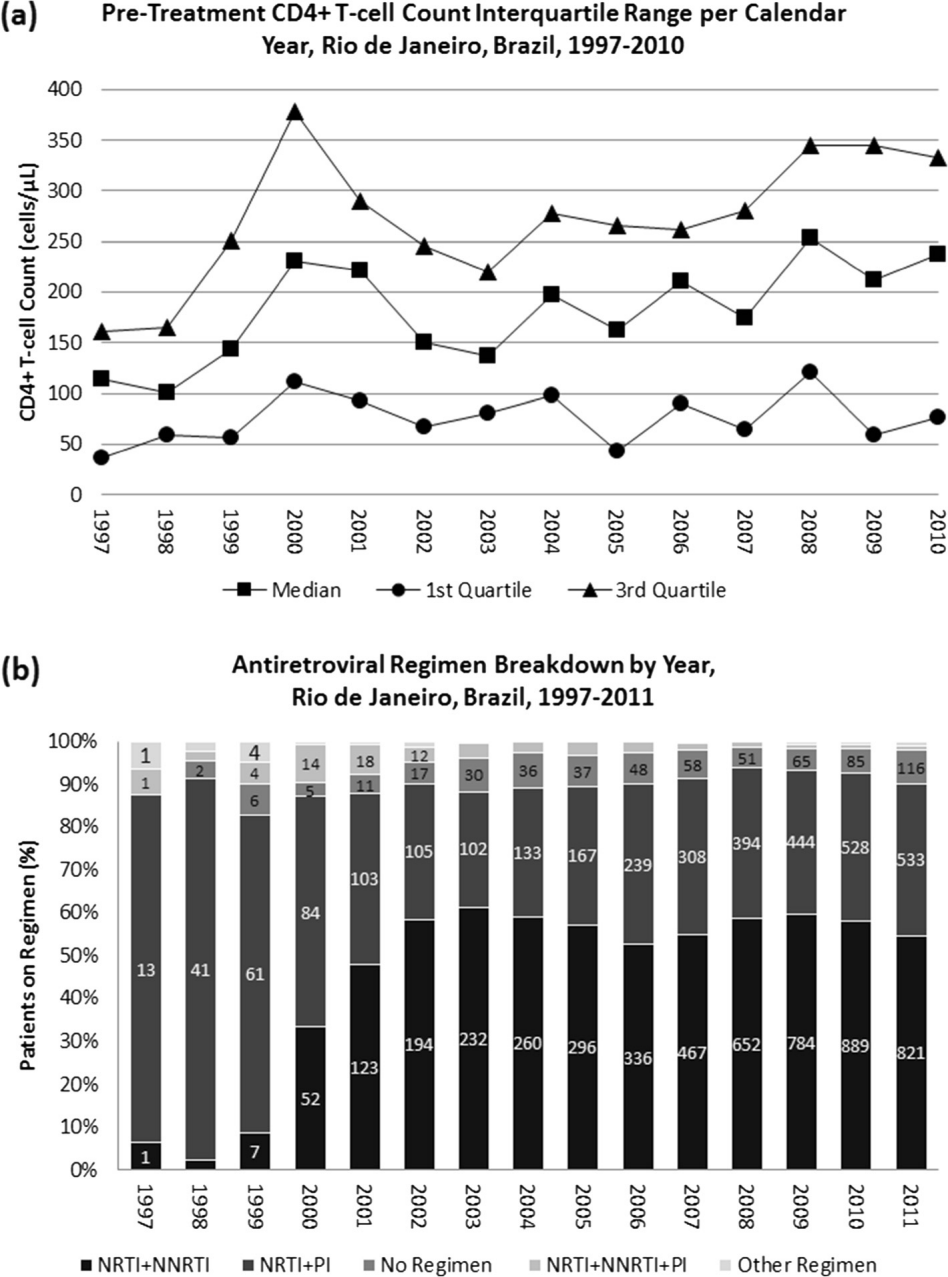
**Trends in (a) pre-treatment CD4 and (b) ART regimen profile per calendar year.** Numbers in columns represent the number of patients prescribed the ART regimen.

Table 1 shows the demographic and clinical characteristics for new patients initiating ART over time. A comparison of the demographic characteristics of new patients included per year showed a significant increase in non-white (p < 0.001) and older patients (p < 0.05), as well as heterosexual men entering the cohort each calendar year (p < 0.001).Figure 1 depicts pre-treatment CD4+ T-cell counts and the primary ART regimens in use per calendar year. Pre-treatment median CD4+ T-cell count significantly increased over the observation period from 114 [37, 161] to 237 [76, 333] cells/μL (p < 0.001). A total of 248 patients did not have a documented pre-treatment CD4+ T-cell count, of which 114 (46%) had a documented acquired immunodeficiency syndrome- (AIDS) defining illness in their clinical chart within the period 90-days prior to 30-days post initiation of ART.The ratio of patients on NNRTI- versus PI-based regimen increased over time, most notably after the year 2000. The number of patients on non-NNRTI- or non-PI-based regimens (depicted as “Other Regimen” in Figure 1) remained stable over time, representing approximately less than 10% of the overall cohort population. The percent of patients with no documented ART regimen in the clinical chart for an entire calendar year (depicted as “No Regimen Documented” Figure 1) varied from 3 to 9% of all patients per year. However, the proportion of patients with an ART interruption of greater than one month duration decreased from 64% in 1997 to 16% in 2011.

**Table 1:** Demographic and clinical characteristics of new patients over time, Rio de Janeiro, Brazil, 1998-2010*

	**1998**		**2000**		**2002**		**2004**		**2006**		**2008**		**2010**		**Overall**		**P-value**
	**N**	**(%)**	**N**	**(%)**	**N**	**(%)**	**N**	**(%)**	**N**	**(%)**	**N**	**(%)**	**N**	**(%)**	**N**	**(%)**	
**New patients**	30	-	79	-	80	-	75	-	136	-	286	-	241	-	1678	-	
**Gender**																	
Male	19	(63)	48	(61)	53	(66)	51	(68)	103	(76)	203	(71)	173	(72)	1163	(69)	0.21
Female	11	(37)	31	(39)	27	(34)	24	(32)	33	(24)	83	(29)	68	(28)	515	(31)	
**Race**																	
White	16	(53)	53	(67)	53	(66)	41	(55)	80	(59)	140	(49)	101	(42)	882	(53)	**< 0.001**
Non-white	14	(47)	26	(33)	27	(34)	34	(45)	56	(41)	146	(51)	140	(58)	796	(47)	
**Age (years)**																	
<30	5	(17)	25	(32)	20	(25)	14	(19)	21	(15)	69	(24)	65	(27)	370	(22)	**<0.01**
30-39	12	(40)	29	(37)	31	(39)	24	(32)	62	(46)	107	(37)	87	(36)	635	(38)	
40-49	11	(37)	17	(22)	19	(24)	28	(37)	32	(24)	76	(27)	63	(26)	468	(28)	
>50	2	(7)	8	(10)	10	(13)	9	(12)	21	(15)	34	(12)	26	(11)	205	(12)	
**Risk group**																	
HT man	4	(13)	13	(16)	25	(31)	21	(28)	38	(28)	75	(26)	56	(23)	429	(26)	**< 0.001**
HT woman	7	(23)	23	(29)	20	(25)	18	(24)	24	(18)	72	(25)	55	(23)	410	(24)	
MSM	10	(33)	31	(39)	21	(26)	21	(28)	48	(35)	103	(36)	87	(36)	554	(33)	
IDU	4	(13)	1	(1)	0	(0)	1	(1)	0	(0)	3	(1)	2	(1)	19	(1)	
Unknown	2	(7)	11	(14)	13	(16)	13	(17)	25	(18)	31	(11)	40	(17)	249	(15)	
Other	3	(10)	0	(0)	1	(1)	1	(1)	1	(1)	2	(1)	1	(0)	17	(1)	
**Education (years)**																	
≤ 8 years	16	(53)	44	(56)	50	(63)	42	(56)	69	(51)	149	(52)	120	(50)	886	(53)	0.40
> 8 years	14	(47)	35	(44)	30	(38)	33	(44)	67	(49)	137	(48)	121	(50)	792	(47)	
**Hep C coinfection**	3	(10)	4	(5)	4	(5)	6	(8)	8	(6)	15	(5)	9	(4)	83	(5)	0.46

*Odd years not shown. Bold text indicates p-value < 0.05. HT, heterosexual; MSM, men who have sex with men; IDU, injecting drug user; Hep, hepatitis.

Out of the 8,770 years of follow-up, there were a total of 2,245 ART interruptions of more than one month duration. The median number of total months of treatment interruption per calendar year was 7 [4, 12]. The proportion of patients with a treatment interruption of more than one month duration trended downward from 64% in 1997 to 16% in 2011. Overall, 74% of patients (1,240/1,678) had at least one year with an ART interruption of more than one month duration, with a median of 2 [1, 5] years with an interruption per patient. The median number of months of follow-up per patient for these patients was 51 [29, 88] compared to 47 [26, 71] months for those who never had an ART interruption of more than one month duration. Demographic characteristics of the patients who never had an ART interruption were overall similar to those who had at least one ART interruption with the exception of there being a greater percentage of IDU in the latter group.

**Figure 2:**
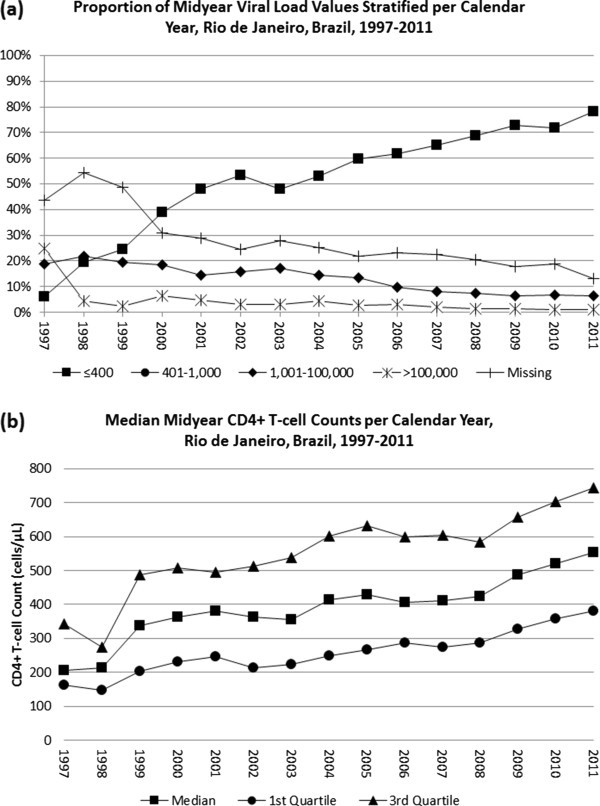
Trends over time in (a) midyear viral load categories and (b) median midyear CD4+ T-cell counts per calendar year for 1,678 individuals.

The median number of viral load measurements per patient per year was 1 [0.8, 1]. Patients contributed a total of 7,278 viral load outcomes. The percentage of patients with a missing viral load outcome decreased from 44% in 1997 to 13% in 2011. Of note, over 78% of patients with a missing viral load outcome had a greater than one month ART interruption documented during the corresponding calendar year.During the observation period, the overall proportion of patients with an undetectable viral load increased from 6% in 1997 to 78% in 2011. Additionally, the median CD4+ T-cell count increased from 207 [162, 343] to 554 [382, 743] cells/μL. The proportion of midyear HIV-1 RNA levels stratified by number of viral copies and median midyear CD4+ T-cell counts per calendar year are depicted in Figure 2.

### Factors associated with undetectable viral load

Table 2 displays the results of the unadjusted and adjusted GEE logistic regression models for having an undetectable viral load. The per-year unadjusted odds ratio (OR) was 1.20 (95% CI =1.17-1.22). After adjusting for clinical and demographic factors, time trends remained significant, with a per-year adjusted OR of 1.18 (95% CI = 1.16-1.21). The following variables within the Risk Group and Pre-Treatment CD4+ categories had significant negative associations with the outcome only in the unadjusted model respectively: Heterosexual Woman, IDU, and Other; and >500 cells/μL and Missing.

**Table 2:** Factors associated with undetectable HIV-RNA level (≤400 copies/mL), Rio de Janeiro, Brazil, 1997-2011

	**Unadjusted OR (95% CI)**	**P-value**	**Adjusted OR (95% CI)**	**P-value**
**Year**	**1.2 (1.17 - 1.22)**	**<0.001**	**1.18 (1.16 - 1.21)**	**<0.001**
**Ethnicity**				
	**White**	1.00 (reference)		1.00 (reference)	
	**Non-white**	0.98 (0.86 - 1.1)	0.70	0.97 (0.84 - 1.11)	0.62
**Risk group**					
	**Heterosexual man**	1.00 (reference)		1.00 (reference)	
	**Heterosexual woman**	**0.83 (0.7 - 0.98)**	**0.03**	0.93 (0.78 - 1.12)	0.46
	**MSM**	1.02 (0.86 - 1.2)	0.85	1.05 (0.87 - 1.27)	0.61
	**IDU**	**0.48 (0.31 - 0.75)**	**<0.01**	0.88 (0.5 - 1.56)	0.66
	**Unknown**	0.99 (0.8 - 1.27)	0.91	1.08 (0.86 - 1.37)	0.50
	**Other**	**0.46 (0.28 - 0.75)**	**<0.001**	0.78 (0.46 - 1.3)	0.34
**Education (years)**					
	**≤8**	1.00 (reference)		1.00 (reference)	
	**>8**	**1.66 (1.46 - 1.88)**	**<0.001**	**1.69 (1.47 - 1.95)**	**<0.001**
Age* (years)					
	**<30**	1,00 (reference)		1.00 (reference)	
	**30-39**	1.14 (0.94 - 1.37)	0.19	1.22 (0.98 - 1.5)	0.07
	**40-49**	**1.55 (1.27 - 1.88)**	**<0.001**	**1.68 (1.34 - 2.11)**	**<0.001**
	**≥50**	**1.98 (1.57 - 2.49)**	**<0.001**	**2.11 (1.61 - 2.77)**	**<0.001**
Years since HIV diagnosis*	**0.98 (0.97 - 1)**	**0.01**	**0.92 (0.91 - 0.94)**	**<0.001**	
**Pre-treatment CD4+**					
	**≤200**	1.00 (reference)		1.00 (reference)	
	**201-350**	1.01 (0.87 - 1.18)	0.87	0.97 (0.82 - 1.14)	0.73
	**351-500**	1.19 (0.95 - 1.5)	0.12	**1.3 (1.02 - 1.64)**	**0.03**
	**>500**	**0.64 (0.49 - 0.83)**	**<0.001**	0.93 (0.7 - 1.24)	0.62
	**Missing**	**0.75 (0.62 - 0.89)**	**<0.001**	0.84 (0.69 - 1.02)	0.08
**Initial regimen**					
	**NRTI + NNRTI**	1.00 (reference)		1.00 (reference)	
	**NRTI + PI**	**0.56 (0.5 - 0.64)**	**<0.001**	**0.72 (0.63 - 0.83)**	**<0.001**
ART interruption*					
	**≤1 month**	1.00 (reference)		1.00 (reference)	
	**>1 month**	**0.29 (0.25 - 0.37)**	**<0.001**	**0.32 (0.27 - 0.38)**	**<0.001**
Hep C coinfection*	1.01 (0.8 - 1.27)	0.95	1.09 (0.84 - 1.42)	0.50	

Results from unadjusted and adjusted logistic regression generalized estimating equations models. A total of 1,492 missing midyear viral load outcomes were excluded from the analysis. OR, odds ratio; CI, confidence interval; MSM, men who have sex with men; IDU, injecting drug user; NRTI, nucleoside reverse transcriptase inhibitor; NNRTI, non-nucleoside reverse transcriptase inhibitor; PI, protease inhibitor; Hep, Hepatitis. *Time-updated variables. Bold text indicates p-value < 0.05.

Older age and more years of formal schooling were positively associated with having an undetectable viral load. ART interruptions >1 month versus ≤1 month per calendar year and initiating a PI-based first-line regimen compared to an NNRTI-based first-line regimen were the factors most strongly negatively associated with having an undetectable viral load. The odds of having an undetectable viral load decreased with each year since HIV diagnosis. Patients with a pre-treatment CD4+ T-cell count between 351 and 500 cells/μL had an increased odds of being undetectable compared to those initiating with a CD4+ T-cell count ≤200 cells/μL.

### Sensitivity analysis

A total of 1,492 missing mid-year viral load outcomes (1,492/8,770; 17%) were excluded from the analysis. Sensitivity analysis assuming all missing mid-year viral load outcomes as detectable did attenuate the overall effect (per-year adjusted OR = 1.14, 95% CI = 1.12-1.16), but did not offset overall time trends or significantly affect the factors associated with undetectable viral load.

## Discussion

In our observational, clinical cohort, significant improvements in virologic outcomes were observed for patients receiving ART over the study period. Between 1997 and 2011, the proportion of patients with an undetectable viral load increased from 7% to 78% and the median CD4+ cell count increased from 207 [162, 343] to 554 [382, 743] cells/μL. Time trends in virologic outcomes were only partially explained by the clinical and demographic factors tested, thus other factors not accounted for in our model, such as improvements in clinical care, treatment options and ART adherence may explain our findings. Our results are consistent with prior studies which suggest that these observed trends in virologic and immunologic outcomes may be partly attributable to improvements in treatment and care [4-10], such as increasing clinician experience [12], improvements in dosing and efficacy of ART regimens, and improved patient knowledge and adherence [1]. In particular, the introduction of newer drugs and drug classes in Brazil, such as raltegravir, darunavir, etravirine and enfuvirtide, within the ART guidelines for salvage therapy, as well as use of resistance testing to guide regimen changes after treatment failure have undoubtedly played a role in the observed improvements over time.

Similar to a prior study of predictors of virologic suppression [5], we found treatment interruptions to be the strongest negatively correlated predictor of having an undetectable viral load. Of note, there was a considerable decrease in the proportion of patients with an ART interruption of greater than one month duration per calendar year. This observed decrease in ART interruptions may be a consequence of improved adherence due to increased patient knowledge and better drug tolerance profiles.

Even though treatment interruptions were assumed from absence of documentation of ART regimen within the patient clinical record and not confirmed with the ARV pharmacy database, it is unlikely that patients with no documented regimen were still retrieving their medications because a physician prescription is required for medication dispensation. Lack of treatment documentation in the clinical chart is therefore likely a surrogate for periods of medication non-adherence or loss-to-follow-up. We did not apply a definition of loss-to-follow-up for excluding patients from the analysis, thus all patients ever initiated on treatment were accounted for until the end of the observation period or documented date of death. The rationale for this was that vital status is exhaustively verified through active patient contact and data linkage with state-wide mortality databases using a previously validated algorithm [13], thus it is unlikely that patients with missing information have died. Given that a large percentage of the patients who did not have an available viral load result also had a treatment interruption during the corresponding calendar year, it may well be that some of these patients were temporarily disconnected from care.

The number of months of follow-up per patient was only slightly higher in the group of patients with at least one versus no ART interruption throughout the study period, thus having no ART interruption is not merely an artifact of differences in follow-up time between the two groups. Given the importance of ART adherence to treatment efficacy [1], development of ART resistance [14], and mortality reduction [15], future research is needed to characterize the reasons for treatment interruptions [16].

We also found older age (≥40 years) [17] and more years of schooling to be associated with increased odds of having an undetectable viral load. This may be attributable to increasing levels of medication non-adherence among younger and less educated patients. Both younger age [18,19] and fewer years of education [18] have been shown to be independently associated with measures of non-adherence. Patients with lower levels of education may have increased difficulty understanding the information provided by the healthcare team, which may negatively impact patient adherence to treatment [20].

Our study was limited by the open cohort design, which is subject to bias both from the inclusion of new patients who may have improved prognoses, as well as the loss of patients with potentially poorer prognoses. We did not conduct a closed cohort analysis, however, a prior study found no difference in time trends for virologic outcomes when comparing a closed versus open cohort [5]. Our results did demonstrate a significant increase in pre-treatment CD4+ T-cell counts per calendar year for patients starting ART, which may partially account for the improving time trends in virologic and immunologic response to ART [21-24]. Our results also reflect the increase in CD4+ T-cell count ART initiation threshold to 350 cells/μL by the Brazilian National AIDS Program in 2006, but also demonstrate that a majority of patients still initiate treatment below the recommended threshold (given that the median pre-treatment CD4+ T-cell count in 2010 was 237) [76, 333]. That is not surprising given that the majority of patients newly presenting for HIV care at our site continue to present with a CD4+ T-cell count <350 cells/μL [25].

Another study limitation was the inability to account for possible viral load blips in our analysis due to the limited quantity of viral load tests performed per patient per year. Despite the fact that Brazilian HIV treatment guidelines stipulate routine viral load and CD4+ T-cell count testing every three to four months for patients on ART, most patients received viral load testing only once per year (median of 1 [0.8, 1] viral load measurement per patient per year).

Of note, patients with a pre-treatment CD4+ T-cell count between 351 and 500 cells/μL were found to have increased odds of having an undetectable viral load in the adjusted model. Having no documented pre-treatment CD4+ T-cell count was negatively associated with our outcome in the unadjusted model. This trend persisted in the adjusted model but achieved only borderline significance (p < 0.10). Patients with missing pre-treatment CD4+ T-cell counts likely initiated treatment based on clinical criteria, and thus were likely to be severely immunosuppressed at the time of treatment initiation. Of the patients with missing pre-treatment CD4+ T-cell counts, 46% had a documented AIDS-defining clinical condition 90-days prior to or 30-days post initiating ART compared to 32% of those with a pre-treatment CD4+ T-cell count.

We were unable to account for the impact of ART regimen changes (both inter- and intra-class drug changes) within our logistical regression model. However, we did find that initiating a PI- versus NNRTI-based first-line regimen was negatively correlated with the likelihood of having an undetectable viral load, consistent with prior research which showed virologic failure was less likely in patients initiating treatment with regimens containing efavirenz versus lopinavir-ritonavir [26]. Of note, lopinavir-ritonavir is the most frequently used PI in our patient population, in accordance with National ART guidelines.

We addressed the possible effects of selection bias due to exclusion of patients with missing viral load outcomes by modeling a worst-case scenario that categorized all missing virologic outcomes as detectable (>400 copies/mL) viral loads. Treating missing outcomes as failures did attenuate the magnitude of the effect, but did not change the overall time trends.

## Conclusions

In conclusion, we found significant improvements in virologic outcomes in a large, urban, publicly run Brazilian clinic from 1997 to 2011 that persisted after adjusting for other factors. This suggests that other unmeasured variables, such as advances in ART dosing and efficacy, for which time is a surrogate, may be contributing to the improvement in HIV-1 viral load suppression. Even though we cannot ensure our results are representative of the entire population of HIV/AIDS patients receiving care nationwide, they are very likely to represent those receiving care in large urban centers in Brazil. Moreover, the increase in the proportion of patients on ART with undetectable viral load levels within our clinical cohort may have implications for the overall community-level reduction of HIV transmission, and should encourage the continued expansion of HIV testing and treatment programs in Brazil.

## Abbreviations

ART: Antiretroviral therapy; CI: Confidence intervals; FIOCRUZ: Oswaldo Cruz foundation; GEE: Generalized estimating equations; HIV: Human immunodeficiency virus; IDU: Injection drug users; IQR: Interquartile ranges; IPEC: Evandro Chagas clinical research institute; MSM: Men who have sex with men; NNRTI: Non-nucleoside reverse transcriptase inhibitor; NRTI: Nucleoside reverse transcriptase inhibitor; PI: Protease inhibitor; OR: Odds ratios.

## Competing interests

JEL has served as a consultant for Merck and Co and GlaxoSmithKline. The authors declare that they have no competing interests.

## Authors’ contributions

DAM: concept development, statistical analysis, manuscript writing. PML: concept development, statistical analysis, manuscript draft revision. JEL: concept development, manuscript draft revision. JLC: concept development, manuscript draft revision. VGV: concept development, data collection, manuscript draft revision. RIM: data collection and management, manuscript draft revision. SWC: concept development, data collection, manuscript draft revision. JDK: concept development, manuscript draft revision. BG: concept development, data collection, manuscript draft revision. All authors read and approved the final manuscript.

## Pre-publication history

The pre-publication history for this paper can be accessed here:

http://www.biomedcentral.com/1471-2334/14/322/prepub
